# Immunotherapies catering to the unmet medical need of cold colorectal cancer

**DOI:** 10.3389/fimmu.2022.1022190

**Published:** 2022-10-05

**Authors:** Jun Yuan, Jiarui Li, Ce Gao, Chun Jiang, Ze Xiang, Jian Wu

**Affiliations:** ^1^ Department of Clinical Laboratory, The Yancheng Clinical College of Xuzhou Medical University, The First People’s Hospital of Yancheng, Yancheng, China; ^2^ Zhejiang University School of Medicine, Hangzhou, China; ^3^ Department of Clinical Laboratory, The Affiliated Suzhou Hospital of Nanjing Medical University, Suzhou Municipal Hospital, Gusu School, Nanjing Medical University, Suzhou, China

**Keywords:** cold colorectal cancer, immune checkpoint inhibitors (ICIs), adoptive cell therapy (ACT), cancer vaccines, cytokines

## Abstract

As a common malignant tumor of gastrointestinal tract, the incidence of colorectal cancer (CRC) has gradually increased in recent years. In western developed countries, it has even become the second largest malignant tumor next to lung cancer. Immunotherapy is a hot topic in the field of cancer therapy, including immune checkpoint blockade (ICB), adoptive cell therapy (ACT), cancer vaccines and cytokines, aiming to improve the ability of the immune system to recognize, target and eliminate cancer cells. However, cold CRC, which accounts for a high proportion of CRC, is not so reactive to it. The development of immunotherapy to prevent cancer cells from forming “immune escape” pathways to the immune system in cold CRC, has been under increasing study attention. There is proof that an organic combination of radiotherapy, chemotherapy, and several immunotherapies can considerably boost the immune system’s capacity to eradicate tumor cells. In this review, we summarized the role of immunotherapy in colorectal cancer. In addition, we propose a breakthrough and strategy to improve the role of immunotherapy in cold CRC based on its characteristics.

## Introduction

As the third most common cancer around the global with a high mortality, colorectal cancer (CRC) generates a negative impact on human public health, and new effective treatment strategies are urgently needed (1, 2). Although CRC morbidity rates have declined with changes in risk factor patterns and the spread of colonoscopy, this progress is increasingly confined to the elderly, the entire population of CRC patients is rapidly becoming younger (3) and its mortality remains high (4), especially in the metastatic CRC which accounts for the vast majority of CRC patients (5).

For the past few decades, immunotherapy, including immune checkpoint blockade (ICB), adoptive cell therapy (ACT), cancer vaccines and cytokines, has attracted wide attention in the field of cancer treatment due to its long-term efficacy in solid tumors such as melanoma and non-small cell lung cancer. Due to the fact that tumors with high tumor mutational burden (TMB) have more immune system-recognizable neoantigens than tumors with lower TMB, high TMB becomes a defining characteristic of immunotherapy response, particularly ICB, in a number of cancer types (6, 7). In the cell genome, DNA sequences called microsatellites are repeated in tandem with a small number of nucleotides, often one to six (8). Mismatch repair (MMR) is a process that removes certain nucleotides from the nascent DNA strand in human cells when they integrate the incorrect nucleotides during DNA replication in order to prevent genetic mutations in progeny cells. The TMB of CRC is closely associated with microsatellite instability (MSI), while MSI is the result of the functional deficiency of the DNA MMR protein (9). A large number of clinical trials have confirmed that the molecular level detected by MSI is highly correlated with the protein level detected by MMR, and the agreement between the two is up to 90% in CRC (10). Mismatch repair deficient (dMMR)/high microsatellite instability (MSI-H) phenotypes accounted for 15% of CRC patients (11), compared with the most of CRC patients presenting proficient mismatch repair (pMMR)/microsatellite stable (MSS) subtype. dMMR/MSI-H CRC has more to do with a higher mutation burden and tumor neoantigen load as well as dense immune cell infiltration compared to pMMR/MSS tumors, and immunotherapy-based therapies have shown strong clinical benefits for this subtype (9, 12). The FDA approved the anti-PD-1 antibody pembrolizumab in 2017 for use in patients with dMMR/MSI-H metastatic colorectal cancer who have progressed after prior therapy and have no satisfactory alternative treatment options. Nivolumab, as well as ipilimumab, also got approval in the treatment of dMMR/MSI-H mCRC patients. In contrast, pMMR/MSS mCRC, commonly known as “cold” cancer, which featuring the infiltration and inflammation of tumor (13), does not respond well to immunotherapy. Therefore, it is important to explore immunotherapies that can benefit such subtype of patients.

This review emphasizes the constraints and problems in providing better care for these patients by summarizing the function of immunotherapies in cold colorectal cancer and outlining their development. In addition, based on the features of cold CRC, we propose a breakthrough and approach to enhance the function of immunotherapy.

## Immune checkpoint inhibitors

The removal of co-inhibitory signals is an approach to breaking the autoimmune tolerance and regulating the efficacy of immune responses. ICB is changing the therapeutic paradigm for many cancers by targeting or blocking the interaction between tumor cells expressing immune checkpoints, thus relieving immune evasion from tumors and restoring the immune function of immune system (14). Several immune checkpoint molecules have been identified as potential targets for immunotherapy. The most widely studied ones now are PD-1, PD-L1, and CTLA-4 (15). In addition, the potential of checkpoints such as LAG-3 (16), TIM-3 (17, 18) and TIGIT (19) in tumor immunity also requires further studying (**Table 1**).

**Table 1:** Protocols to improve the efficacy of immune checkpoint blockade in cold colorectal cancer.

Target	Compounds	Model	Outcome	References
PD-1	Anti-CTLA-4	CT26.WT murine colorectal cell line	Significantly inhibiting tumor growth compared with monotherapy Enhancing expression of TIM-3	(20)
PD-1	Anti-CTLA-4	21 pMMR and 2 dMMR patients	Increasing percentage of immune checkpoint expression in CD8^+^ cell Enhancing RFS of some patients	(21)
PD-L1	Anti-TIGIT	13 MSS and 3 MSI-H colorectal tumor cells	Improving the functional qualities of TILs	(19)
PD-1	Anti-TIM-3	CD8+ T lymphocytes isolated from patients with CRC	Increasing the frequency of interferon-γ and tumor necrosis factor-α Promoting the proliferation of tumor antigen-specific CD8^+^ T cells	(22)
PD-1	FOLFOX	CT26 and MC38 colon carcinoma cell lines	Inducing complete and durable tumor cure Upregulating the immune checkpoint expression	(23)
PD-1 CTLA-4	Temozolomide	MGMT-silenced MSS mCRC patients	Enhancing the tumor mutation burden Improving PFS, OS and ORR	(24)
PD-L1	Cetuximab	71 MSS and 3 MSI-H RAS wild-type mCRC patients	11.6 months of mOS, 3.6 months of mFPS and ORR of 8.5% in MSS CRC patients	(25)
PD-1	Regorafenib	24 MSS and 1 MSI-H CRC patients 25 GC patients	Enhancing ORR Increasing response rate to PD-1 blockade	(26)
PD-L1	Cobimetinib	84 patients with mCRC	Enhancing the tumor mutation burden Increasing infiltration of CD8^+^ T cell	(27)
PD-1	Radiotherapy	22 patients with pMMR mCRC	Enhancing the anti-tumor immune activity	(28)
PD-L1 CTLA-4	Radiotherapy	24 patients with chemotherapy-refractory pMMR mCRC	11.4 months of mOS, 1.8 months of mFPS and ORR of 8.3% Promoting the proliferation of tumor antigen-specific CD8^+^ T cells	(29)

In a phase II clinical trial higher progression-free survival (PFS) and overall survival (OS) rates were found in MSI-H patients using pembrolizumab (30). While in another phase II clinical trial (NCT01876511), 41 patients with MSS/MSI-H advanced mCRC received pembrolizumab intravenously. The objective response rate (ORR) and OS rate in the cohort of patients with MSI-H mCRC was 40% and 78%, compared with 0% and 11% in patients with MSS mCRC (31). The response of these two phenotypes of CRC to ICB showed a dramatic difference. The resistance mechanisms implicated against PD-1 immune checkpoint blockade in MSS CRC include loss of antigen presentation, abnormal cell signal transduction, and immunosuppression (32). MSI-H tumors have higher TMB, more neoantigens, and higher levels of multiple checkpoints expression (33), which may partly explain why immune checkpoint blockade therapy is more effective in MSI-H CRC than in MSS CRC. The resistance to PD-1/PD-L1 may associate with the presence of liver metastases as well, which are related to poorer PFS and OS (34). In a retrospective study, PD-1/PD-L1 inhibition showed a clinical advantage in MSS CRC patients without liver metastasis, bringing new inspiration to MSS colorectal cancer treatment (35).

### Combination of two ICIs

The combination of PD-1/PD-L1 and CTLA-4-blocking antibodies may have a controllable safety profile and inspiring antitumor activity in MSS/pMMR colorectal cancer patients (36). A total of 30 mice were divided into three treatment groups in an experiment using the mouse colorectal cancer model CT26: untreated, anti-PD-1 antibody monotherapy, or anti-PD-1 and anti-CTLA-4 antibody combination (DICB). The findings demonstrated that, in comparison to the other two groups, DICB might dramatically restrain tumor progress. Nonetheless, tumor growth was not stopped or reversed in the DICB group (20). Durvalumab and tremelimumab were applied to 21 pMMR and two dMMR resectable liver metastasis colorectal cancer in a clinical trial (NCT02754856). Two dMMR patients showed complete pathological response, and evidence of T cell activation as well as prolonged RFS was observed in pMMR mCRC patients (21).

Cell immune responses are essential for tumor growth in colorectal cancer. Cytotoxic anti-tumor-specific CD8^+^ T cells are present in primary tumors or in metastatic tumor sites and can specifically recognize and kill autologous cancer cells (37). Tumor immune-infiltrating cells (TIL), especially CD8^+^ T cells, are one of the most effective prognostic parameters for both local and metastatic colorectal cancer (38). MSI-H/dMMR CRC has a low probability of distant metastasis, while the proportion of tumors potentially responding to immunotherapy in patients with pMMR/MSS CRC is significantly increased in metastases (39). The MSS CRC patients had a reduced CD8^+^ TIL percentage as compared to the MSI-H CRC patients, respectively (40, 41). TIGIT and TIM-3 are coinhibitory receptors on the surface of CD8^+^ T lymphocytes that inhibit CD8^+^ T cells secreting cytokines and promote CD8^+^ T cell depletion, providing insight for the combined blockade of ICIs (22, 42). In one study, atezolizumab monotherapy and the combination of atezolizumab and tiragolumab were used in 13 MSS CRC patients (19). The data suggested that atezolizumab alone can only induce the activation of the immune response in tumor infiltrating lymphocytes (TILs) in MSI tumors, while the combination therapy of atezolizumab plus tiragolumab was able to reactive CD8^+^ TILs in 46% of MSS CRC patients. Liu et al. found that blockage of the Tim-3 and PD-1 pathways enhanced the frequency of proliferating tumor antigen-specific CD8^+^ T cells, achieving a reversal of tumor-induced T cell exhaustion/dysfunction in colorectal cancer patients (22).

### Combination with chemotherapy

Increasing evidence suggests that chemotherapy can activate the immune system either by directly stimulating anti-tumor responses or by modifying local immune microenvironment (43, 44), providing a strategy to couple chemotherapy with ICIs to transform the so-called “cold” non-infiltrated tumors into “hot” highly infiltrated tumors.

The combination chemotherapy of leucovorin, fluorouracil and oxaliplatin (FOLFOX) is often used in the first-line treatment of mCRC (45, 46). Meanwhile, FOLFOX causes immunogenic cell death and the recruitment of antigen-presenting immune cells, and its role in anti-tumor immune infiltration cannot be underestimated (47). Based on this theory, Dosset et al. adapting FOLFOX with or without anti-PD-1 therapy to CT26 tumor-bearing BALB/c mice showed that FOLFOX restores the immunological environment to sensitize colorectal cancer to ICIs and only the FOLFOX/anti-PD-1 group of mice achieved a complete cure of cancer without recurrence (23). This provided a new perspective for the treatment of MSS CRC. A Phase Ib/II study (NCT03202758) evaluated how the combination of durvalumab, tremelimumab and FOLFOX worked in MSS tumors with RAS mutated status (24). The results showed a RFS rate of 95% and a PFS of 70.7% was expected. FOLFOX is therefore a potent adjunct to ICB. More fully designed clinical trials are still needed to determine the optimal dosing regimen to maximize the immunogenicity of the FOLFOX.

Temozolomide is an alkylating agent approved for use in patients with glioblastoma, which works by causing cross-linking between or within strands of DNA, destabilizing DNA during replication (48). This provides the rationale for inducing the immunosensitization of pMMR/MSS mCRC, which is MGMT silenced, by temozolomide initiation. A multicenter, single-arm phase II trial (NCT03832621), whose first phase of oral temozolomide 150 mg/sqm treatment in MSS mCRC, and a second phase of combination therapy with ipilimumab 1 mg/kg and nivolumab when no progression was observed, had the primary endpoint being the 8-month PFS rate calculated from the enrollment of patients starting the second treatment portion (49). 24% of the patients entering the second part of treatment showed a PFS rate of 36%. The results demonstrated that the combination treatment of temozolomide with ipilimumab and nivolumab may produce a long-lasting clinical benefit in the MSS mCRC.

### Combination with anti-angiogenic agents

Angiogenesis, which is closely linked to tumor progress, is one of the hallmarks of cancer (50). Therefore, inhibiting angiogenesis is a popular treatment for cancer (51). VEGF (vascular endothelial growth factor) regulates the progress of tumor angiogenesis (52), and is currently the only known angiogenic factor continuously expressed throughout the tumor life cycle. There are two main classes of antitumor drugs acting on the VEGF-VEGFRs pathway. One is monoclonal antibodies, and the other is small molecule VEGFRs tyrosine kinase inhibitors.

Lee et al. demonstrated that the combination of avelumab and cetuximab (targeting EGFR) showed an ideal 11.6-months median OS and a longer median PFS in MSS mCRC (25).While in a phase Ib trial REGONIVO (NCT03406871), 24 pMMR/MSS CRC patients received the treatment of regorafenib (VEGFRs tyrosine kinase inhibitor) plus nivolumab (26). The median PFS for this treatment was 7.9 months, and the ORR was 36%. This demonstrated that in pMMR/MSS CRC patients, the combination of regorafenib and nivolumab had a manageable safety profile and increased antitumor efficacy. However, the encouraging results of the REGONIVO were not reproduced in the following series of clinical practice. A phase I/Ib study (NCT03712943) treated 52 pMMR CRC patients with a combination of regorafenib and nivolumab (53).Only 10% of the patients had partial remission, 2.5% had confirmed remission, 53% were stable with a disease control rate of 63%, showing a limited anticancer activity of this therapy in pMMR CRC. This result could be explained by the differences in the patient populations of the two study. This cohort recruited more patients with mCRC than the REGONIVO trial. Former study has proved that metastasis was often accompanied by diminished efficacy of ICB in multiple malignancies (54). Therefore, further studies are required to validate the efficacy of the combination therapy of ICIs with anti-angiogenic agents in cold CRC.

### Combination of MEK inhibitor

Underloading of tumor antigens impairs the T-cell-mediated immune response and improving the antigenicity of tumor is a feasible strategy to target cold CRC. MEK signaling pathway is one of the most classic signaling pathways in the tumor field whose protein overexpression or mutation has been found in many malignant tumors. The signaling molecule, MEK, is a key intermediate in the MAPK pathway (54). The MAPK axis is essential in the proliferation and apoptosis of CD8^+^ TIL (55–57). Preclinical models demonstrated that highly selective MEK inhibitors inhibited tumor progress and promoted changes in the proliferation and effector phenotype of CD8^+^ TILs (55).This showed that the MEK inhibitors combined with ICIs treatment may synergistically inhibit tumor growth.

A phase I/Ib trial (NCT01988896) applied the combination of the MEK inhibitor cobimetinib and the anti-PD-L1 antibody atezolizumab to patients with solid tumors (27). Of the 84 mCRC patients enrolled, a total of six MSS patients and one MSI-H patient showed a clinical response, with an ORR of 10% and a median OS of 10 months, demonstrating the safety and tolerability of this therapy. Based on these results, cobimetinib was applied to MSS CRC patients in combination with atezolizumab in the subsequent phase III study (NCT02788279) (46). Unfortunately, although atezolizumab plus cobimetinib improved OS compared to atezolizumab monotherapy, the efficacy of the combination was inferior than regorafenib.

### Combination of radiotherapy

Radiotherapy is also an ideal option to promote T cell infiltration into cold tumor. RT can release neoantigens and inflammatory cytokines during treatment and has the ability to increase CD8+ cytotoxic T cells, thus regulating the TME and stimulating immune system response (58, 59). Grapin et al. proved that the combination of ICIs and RT had the value of further clinical studies (60). And another preclinical model combined DNA repair inhibitors, RT, with anti-CTLA-4, which was a multimodal treatment regimen that reduced dosing, potentially inhibiting chemical resistance and dose-limiting toxicity (61). The combination therapy of RT and ICB exhibited great prospects in the treatment of refractory CRC and should be intensively studied urgently.

A non-randomized phase II study (NCT02437071) was designed to evaluate the combination of pembrolizumab with RT in patients with pMMR mCRC (28). Although only one patient out of the 22 patients achieved an objective response in the unirradiated area, it still gave us inspiration of further investigation. A large number of studies centered on combination of radiotherapy and immunotherapy are ongoing. Segal et al. recruited 24 patients with chemotherapy-refractory pMMR mCRC and applied durvalumab, tremelimumab and radiotherapy (29). Two patients treated had an objective response in the unirradiated tumors, with an ORR of 8.3%. 23 patients remained progression-free at 12 months after PR. The best response in the three patients (12%) was a stable disease, with no SD being observed for 4 months or longer. To determine the combination of radiotherapy and immunotherapy is challenging but crucial in clinical trials.

## Adoptive cellular therapy

ACT is an emerging immunotherapy and is a rapidly developing field of clinical research. Compared with traditional methods, ACT has the advantages of high specificity, short acting time and less interference by internal factors. By isolating the immunoactive cells from the tumor patients, they were expanded and modified *in vitro*, and finally returned to the patients, so as to trigger passive or active immunity (62). There are currently four major ACT approaches: chimeric antigen receptor (CAR) T cells, genetically engineered T cell receptor (TCR), tumor-infiltrating lymphocytes (TILs), and cytokine-induced killer (CIK) cells, the latter three of which are still in the early stages of research compared to CAR-T. ACT has achieved remarkable results in hematological malignancies, such as acute B-cell leukemia and multiple myeloma (63, 64). However, the role in solid tumors is still unclear, and its therapeutic efficacy and safety remain to be verified in clinical studies (**Table 2**).

**Table 2:** Different applications of ACT in colorectal cancer.

Approach	Model	Outcome	References
CEA CAR-T	10 refractory CRC patients with liver and lung metastasis	Decreasing expression level of CEA Decreasing tumor size	(65)
CEA CAR-T IL-10	38 MSS CRC patients with liver metastases	Inhibiting exhaustion of CD8^+^ T cell Increasing cytotoxicity of CEA specific CAR-T cell	(66)
NKG2D CAR-T	LS174T and HCT-116 human colorectal cell lines	Increasing abundance of CD4^+^ cells Improving anti-tumor ability of CAR-T cells *in vitro* and vivo	(67)
HER2 CAR-T	Xenograft CRC tumor models	Potent and specific cytotoxicity against CRC cells Significantly inhibiting tumor growth and migration Prolonging ORS of mice	(68)
KRAS G12D-specific CD8^+^ TILs	Patient with MSS mCRC	A sustained resolution of the symptoms Enhancing regression of lung metastases	(69)
CIK cells Chemotherapy	60 patients with CRC	Increasing infiltration of CD8^+^ T cells Improving PFS and OS Reducing recurrence rate of CRC	(70)
DC-CIK cells Chemotherapy	Patient with advanced CRC	Improving PFS and OS significantly Alleviating immune suppression Promoting proinflammatory cytokines	(71)

### Chimeric antigen receptor T-cell and genetically engineered T-cell receptor

T cells are genetically modified to express a specialized chimeric immune-receptor, enabling T cells to identify oncogenic antigens, targeting specific proteins, in an MHC-independent manner (72, 73). CAR-T is currently prevailing for its applicability to all subtypes of colorectal cancer and its capacity to overcome the barrier of cold tumor insensitivity to immunotherapy. One of the difficulties in the development of effective ACT in solid tumors, specifically targeting CAR-T cells, is that CAR-T can produce toxic side effects in healthy tissues. Targeting tumor cells and avoiding target recognition in normal human tissues to overcome target antigen heterogeneity has been a key challenge in the development of solid malignant tumor cell therapy (74). Current targets used for anti-CRC CAR-T cell therapy are generally not exclusive to tumor cells, which can sometimes lead to targeted external tumor toxicity (75). Therefore, CAR-T cell therapy suitable for CRC needs to be further explored.

Carcincoembryonic antigen (CEA) is the most frequently studied target for CAR-T cells for the treatment of CRC. In a phase I clinical trial (NCT02349724) of CEA CAR-T treatment in CEA^+^ CRC patients, 10 patients with relapsed and refractory CRC metastasis were enrolled (65). The results showed that 70% of the patients were stable after treatment, with a slightly reduced tumor diameter, and 20% were stable for more than 30 weeks. Discussion of the mechanisms of immune evasion is vital for the development of new approach to overcoming drug resistance. In a study using an electrostatic copy model in nude mice, it was confirmed that rhIL-12 (recombinant human IL-12) enhanced the anti-neoplastic capacity of CEA CAR-T cells (76). Another study of combined treatment with neutralizing antibodies against IL-10 and CAR-T in 38 patients with MSS liver metastatic CRC showed that IL-10 increased CEA-specific CAR-T cell activation and promoted CAR-T mediated tumor cell death, inducing nearly 70% apoptosis in tumor biopsies (66). These results also inspired us to bind cytokines to CAR-T cells is a feasible strategy.

NKG2DL, EGFR, and HER2 are also suitable candidates for CAR-T targeting. Transduction of NKG2D CAR-T cells from non-viral third-generation NKG2D CAR cells had a significant inhibitory impact on tumor progress in mice (67). HER2 CAR-T cells exhibited antitumor activity in CRC xenograft models, and some tumors were even completely eliminated. Since HER2 levels are significantly higher in mCRC than in primary CRC, HER2 CAR-T cells have an effective immunotherapy capacity for Mcrc (68). No occurrence of adverse reactions was observed in any of the above studies, indicating the safety of the above therapies.

The genetic alteration of T-cell receptors to enhance their capacity to recognize and kill certain cancer cell antigens is a trait shared by CAR-T and TCR-T technologies, albeit their precise approaches vary. Unlike TCR, which depends on MHC processing, CAR attaches to tumor surface antigens, but the spectrum of tumor-specific antigens that TCR-T cells may detect includes certain intracellular antigens (77). TCR is currently the most likely T cell immunotherapy to make a breakthrough in solid tumors. The search for immunogenic neoantigens is also crucial for TCR-T cells. The limitation of CEA as a target for CRC may be seen in the fact that TCR-T cells targeting CEA not only produced tumor regression but also severe colitis and other problems (78). In current clinical studies conducted both domestically and internationally, TCR-T cells directed against NY-ESO-1 have demonstrated high safety and effectiveness in the treatment of refractory recurrent melanoma, synovial sarcoma, multiple myeloma, lung cancer, and other cancers (79). Ny-eso-1-specific TCR-T cells showed strong anti-tumor ability in MSS CRC cell line and significantly prolonged the survival of mice while decitabine treatment can synergistically enhance the specific killing ability of TCR-T cells by upregulating the expression of NY-ESO-1 (80). Another great target for melanoma treatment is cancer-testicular (CT) antigen. But its applicability in CRC is constrained by the lack of its expression. The hypomethylating agent 5-AZa-2 ‘-deoxycytidine (DAC) induces the expression of CT in CRC and makes tumor cells more sensitive to TCR-T cells (81). Furthermore, several targets, such the PCSK9, LDLR, and others have a role in regulating TCR signaling in CD8+T cells *via* a variety of mechanisms (82). Regrettably, TCR-T clinical trials for CRC are still in the early stages of development, and more research is required to fully understand its therapeutic potential.

### Tumor-infiltrating lymphocytes and cytokine-induced killer cells

TILs are the lymphocytes that leave the blood flow and enter the tumor, which is a polyclonal and heterogeneous population of cells, including lymphocytes (T cells, B cells, and natural killer (NK) cells), macrophages, dendritic cells, and neutrophils, with extensive antigen-recognition capabilities in tumor cells (83). Studies have identified associations between TIL load, mutation rate, and the immune landscape of CRC patients and clinical outcomes (15, 84). TIL within the CRC is beneficial for patient survival and can serve as a prognostic indicator.

Although the TILs have been applied in varieties of cancers, only a few trials of the TILs have been performed against the CRC. Gardini et al. found that TILs combined with high-dose IL-2 showed no distinct difference in clinical outcome compared to conventional chemotherapy (85). In another trial (NCT01174121), researchers extended the KRAS G12D-specific CD8^+^ T cell clone and reinfused the TILs into the patients, and observed a sustained resolution of the symptoms, with six out of seven lung metastases eradicated. This result suggested that ACT targeting TIL cells of tumor neoantigens was an attractive therapeutic option for MSS CRC tumors with low mutational burden (69).

In general, the pMMR/MSI-L/MSS CRC has a lower TMB and limited amount of TILs (86) which is difficult to collect and amplify, and cannot meet the requirements of ACT (87). Moreover, TIL products need to be customized for individual patients, which is time-consuming and costly (88). Therefore, new improvement schemes are still being explored. As an alternative source of TILs, CIK cells are a heterogeneous group of cells co-cultured by human peripheral blood mononuclear cells (PBMCs) with various cytokines (89) which have a similar function to that of NK cells (90). A randomized controlled trial aimed to evaluate the effect of autologous CIK cells immunotherapy on OS and PFS in patients with colorectal cancer. The median PFS and median OS in the CIK group were 25.8 and 41.3 months, compared to 12.0 and 30.8 months in the control group (70). Another study reviewed 142 advanced colorectal cancers treated with conventional or adjuvant DC-CIK, and analyzed their respective 1-, 3-, and 5-year OS rates and PFS rates (71). The 5-year PFS and OS rates in the DC-CIK group were 57.4% and 41.3%, while the 5-year PFS and OS rates in the non-DC-CIK group were 33.6% and 19.4%, respectively. The data suggested that DC-CIK cells used as adjuvant therapy in combination with first-line therapy can significantly reduce the mortality and recurrence rate of advanced colorectal cancer.

## Cancer vaccines

**Figure 1:**
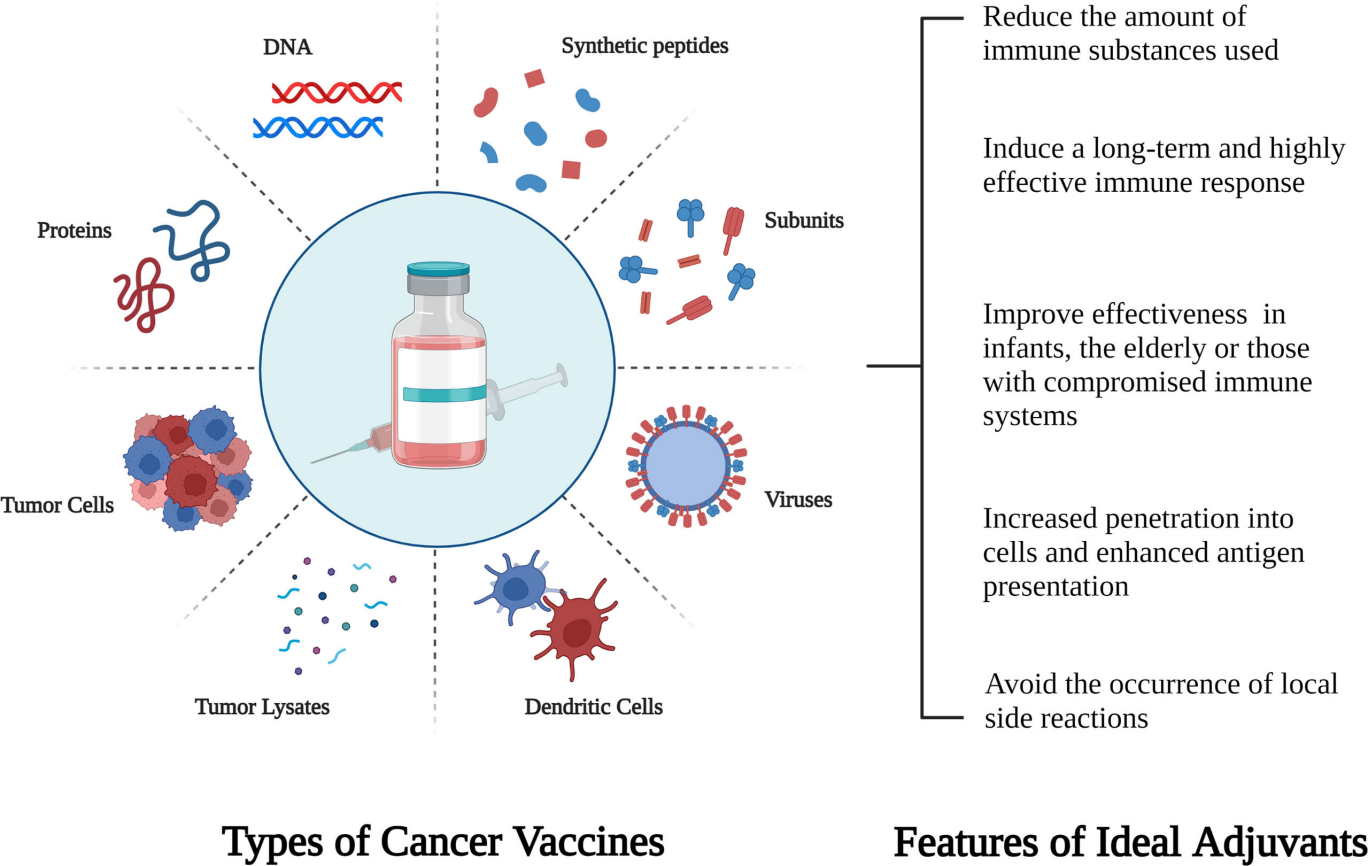
Schematic diagram of different types of cancer vaccines and characteristics of new adjuvants.

One of the key factors or processes to be tackled to achieve clinical benefit in cold CRC is to trigger immune response, while cancer vaccine is an ideal immunotherapy strategy. The most critical step in designing a cancer vaccine is to find the right antigen. Tumor antigens are traditionally classified into tumor-associated antigens (TAA) and tumor-specific antigens (TSA). Nowadays, the principle of most therapeutic cancer vaccine is based on the establishment of TAA -specific antitumor immune response to eliminate tumor cells expressing these antigens (91). Cancer vaccines include cell (autologous, DC) vaccines, protein/peptide vaccines, and gene vaccines (92) (**Figure 1**). Many studies have identified a variety of TAA expressed by CRC cells as potential targets for vaccine immunotherapy, including CEA, WT1, MUC1, RNF43, GUCY2C, SART3, and hTERT (91). The three targets that are the most comprehensive and widespread are CEA (93),enteric in guanylyl cyclase 2C (GUUCY2C) (94) and melanoma-associated antigen (MAGE) (95).

CEA is less frequently expressed in intestinal epithelial cells but is overexpressed in CRC cells. Nonetheless, CEA is poorly immunogenic as an “autoantigen”, and although many methods have been used to vaccinate against CEA, none of the earlier reported trials have produced objective responses (96). Allosteric peptide ligand (APL) is a strategy to solve this problem. A phase I clinical trial was immunized with the amplified DCs loaded with allosteric peptide ligands derived from the CEA (97) and two out of the 12 patients had stable disease. A patient with progressive mCRC had completely eliminated lung metastasis and malignant pleural effusion. Evidence indicates that anticancer chemotherapeutic agents may in fact stimulate the vaccine-induced immune cell responses through its antigenicity and adjuvanticity (98). A phase II clinical trial tested the efficacy of the CEA-specific T cell response initiated by the ALVAC (Canary pox virus) vaccine expressing CEA and B7.1 combined with FOLFIRI chemotherapy in metastatic colorectal cancer (99). Large increases in T cell levels were observed in some patients. Of the 104 evaluable patients, the ORR was 40.4%, with two complete responses, and another 37.5% were in a stable condition.

Cancer vaccines against individual TAA have limited effects, and the development of vaccines containing multiple TAA-derived peptides or targeting multiple TAA’s is underway. In a phase I clinical trial, patients received a combination of a novel peptide vaccine derived from RNF43 (ring finger protein 43) and TOMM34 (34-kDa translocase in the outer mitochondrial membrane) and chemotherapy (100). Of the 21 evaluable patients, 95% had enhanced specific CD8^+^ T cell infiltration, and 83% were under control. The recently reported recombinant poxvirus vaccine against MUC1 and CEA (BN-CV301) applied in 12 patients (NCT02840994) showed high efficiency in producing antigen-specific T cells directed against MUC1 and CEA (101). Single-agent BN-CV301 produced a partial response (PR) in one patient, and the disease stabilization period was prolonged in multiple patients.

Apart from relying on TAA, vaccine-induced immune reactivity to antigens depends on the use of adjuvants, key components that enhance antigen-specific immune response. One study evaluated the efficacy of the WT colon tumor cell antigen vaccine used with recombinant mouse GM-CSF and IL-2 as cytokine adjuvants in a BALB/c murine tumor model (102). The results showed that the combination effectively activated the autologous T cell response, prolonged survival and significantly inhibited tumor growth, compared to the adjuvant or the inactivated antigen alone. Numerous studies have shown that CpG, OX40 agonist, and anti-PD-1/PD-L1 antibodies can be used as adjuvants to improve vaccine activity through different mechanisms. It was newly explored that how the extent to which different combinations of local injected antibodies to CpG ODN, anti-PD-1, and OX40 agonists improved the efficacy of oncolytic vaccinia-virus (VVs) vaccines armed against IL-2 (103). The results showed that dual treatment with vvDD-mIL2 and CpG injection resulted in specific CD4^+^ and CD8^+^ T cells response, weakening immune suppression. PD-1 blockade also significantly promoted the antitumor activity.

## Cytokines

Since cytokines are important regulatory components of TME, cytokine-based immunotherapy is a promising field in cancer therapy. Cytokines are soluble proteins which mediate cell-to-cell communication and can interfere with cell cycle in different cell types (104). As molecular messengers of immune responses, cytokines are able to modulate the host immune responses to cancer cells, including T-cell initiation and activation, and effector T-cell infiltration at cancer sites (105, 106). Cytokines can also be targeted to bind to membrane receptors, and then directly affect carcinogenesis by altering tumor phenotype (104). So far, there are more than 130 different functional cytokines, of which only two, IFN-α and IL-2, have received FDA approval (107). Cold CRC is characterized by low immune infiltration, and the function of cytokines to expand the proliferation of immune cells and induce the recruitment of immune cells is an ideal method to transform cold tumors into hot tumors (**Table 3**).

**Table 3:** Different applications of cytokines in colorectal cancer.

**Approaches**	**Model**	**Outcome**	**References**
IFN-α2 Anti-VEFGR2	HCT-116 and SW620 human CRC cell lines HCT-116-bearing NOD-SCID mice model	Inhibiting migration and invasion of tumor Arresting the cell cycle of CRC cells Enhancing antitumor activity of DC cells	(108)
IFNα-mutant Anti-VEFGR2	HCT-116 and SW620 human CRC cell lines CHO-pro hamster ovary cell line HL-7702 human liver cell line HCT-116-bearing BALB/c mice model	Enhancing ability to induce apoptosis in CRC cells Promoting DC cell maturation and enhancing antitumor activity Improving tumor microenvironment and inhibiting tumor growth and migration	(109)
IL-2 IL-15 cetuximab	52 patients with CRC	Activating exhausted NK cells Improving tumor killing ability of CRC-NK cells	(110)
IL-2 oxaliplatin plus 5-fluorouracil	mCRC patients with or without pretreatment lymphocytopenia	Enhancing efficacy of chemotherapy in patients with pretreatment lymphocytopenia Improving immune status	(111)
IL-21	C57BL/6J and C57BL/6J^-ApcMin^ mice model B6.129S-Il21^tm1Lex/Mmucd^ mice model	Increasing T cell and NK cell infiltration Inhibiting the polarization of lymphocytes toward the Th17 phenotype	(112)

### Interferons

Interferon is of great significance for the development of novel antitumor therapies. IFNs are divided into three types based on their function and target receptors: type I (α, β, ϵ, κ and ω), type II (γ) and type III (λ) (113).

Loss of IFN-I signaling is a typical characteristic of noninvasive tumors. As one of the markers of hot tumors in TME, IFN- I, including IFN-α and IFN-β, plays an important role in cancer antigen presentation by activating a variety of immune cells and upregulating MHC class I surface molecules (114). Because IFN-α shows effective antiangiogenic activity (115), the combination of the anti-VEGF antibody bevacizumab with IFN-α was approved by the FDA as a first-line treatment for metastatic renal cell carcinoma in 2009 (116). Fusing IFN-α2 with anti-VEGFR2 significantly inhibited the proliferation, migration and invasion of CRC cells, and promoted the apoptosis and cell cycle arrest of CRC cells (108). However, the affinity of IFN-α2 for its receptor and its direct cytotoxicity were decreased in this combination. Thus, subsequent studies mutated IFN-α to further improve the anti-tumor efficacy and regulate TME more effectively by promoting dendritic cell maturation and enhancing CD8^+^ T cell infiltration (109). Although there is *in vitro* evidence that IFN-β inhibits tumor cell proliferation more effectively than IFN-α, no clinical trials have demonstrated its efficacy in cancer therapy (105). Additional preclinical and clinical research is required to examine more potent combo therapies.

As the only TFN-II, IFN-γ is a pleiotropic cytokine both coordinating pro-tumor and anti-tumor immunity in the tumor microenvironment (117). Cell cycle inhibition can greatly enhance the pro-apoptotic function of IFN-γ (118). IFN-γ has been shown to selectively eradicate label-preserving cancer cells (LRCC), a group of stem-like cancer cells that exhibit slow proliferation, enhanced chemical resistance, and tumor-initiation (119). Another study found increased PD-L1 expression through JAK2/STAT1 signaling after IFN-γ stimulation, which inhibited the antitumor immune response (120). However, IFN-γ gene signatures can be used as predictive markers of clinical response to ICIs (121). Increased IFN-γ concentrations are associated with better ICB efficacy, and the combination of ICIs and IFN-γ may have additional value in antitumor effects. The complex role of IFN-γ in TME remains to be studied in order to enhance its antitumor effect and limit its tumor-promoting effect.

### Interleukins (ILs)

The use of cytokines derived from the IL-2 family, such as interleukin IL-2, IL-7, IL-15, and IL-21, to stimulate the anti-tumor response is prevailing in the field of immunotherapy (122). Among them, IL-2, IL-15, and IL-21 are also the most widely studied subjects, with different effects on CD8^+^ T cells, NK cells and Tregs (123, 124). IL-2 can induce preferential activation of Tregs and stimulate the expansion of CD8^+^ T cells (125). Nevertheless, continuous exposure to IL-2 results in T cell hyperactivation and is prone to apoptosis. IL-15 and IL-21 have been shown to be superior to IL-2 in the development and maintenance of NK cells, thus protecting them from apoptosis (126). Interleukins, as monotherapy or combined with other biological agents, are actively pursued in the clinical studies.

IL-2 is one of the cytokines approved by the FDA for the treatment of metastatic RCC and metastatic melanoma. A study showing that therapeutic strategies combining cetuximab, IL-2, and IL-15 could activate phenotypic and dysfunctional blood NK cells and improve cytotoxicity in CRC patients provided novel insights into CRC therapies based on ILs (110). Another study demonstrated that IL-2 pretreatment could promote lymphocyte proliferation and enhance the efficacy of oxaliplatin plus 5-fluorouracil chemotherapy in mCRC patients (111).

IL-15 has attracted attention as a potential therapeutic agent in CRC immunotherapy. A preclinical model demonstrated that IL-15 deficiency increased tumor burden due to NK and CD8 + T cell immunodeficiency as well as the inflammatory environment supporting tumors, suggesting that intestinal homeostasis and inhibiting inflammation-induced tumorigenesis were dependent on IL-15 (127). Another study showed that IL-15 which was either administered alone in CRC rats or in combination with leucovorin reduced chemotherapy-induced gastrointestinal toxicity and enhanced the antitumor activity of 5-fluorouracil (128).

IL-21 has both tumor-promoting and tumor-suppressive effects. Elevated IL-21 expression levels were detected in the CRC microenvironment whose result indicating that IL-21 levels were inversely correlated with poor survival (129). However, another study proved that IL-21 stimulated a cytotoxic anti-tumor response in CRC, and the lack of IL-21 promoted intestinal tumor formation through the dysregulation of the Th1/Th17 axis (112). A phase I trial tested the safety and tolerability of a recombinant IL-21 (rIL-21) therapy combined with cetuximab in stage IV CRC (130). The results showed rIL-21 plus cetuximab was well tolerated at doses up to 100g kg (–1) and led to increased expression of immune markers.

## Conclusions

**Figure 2:**
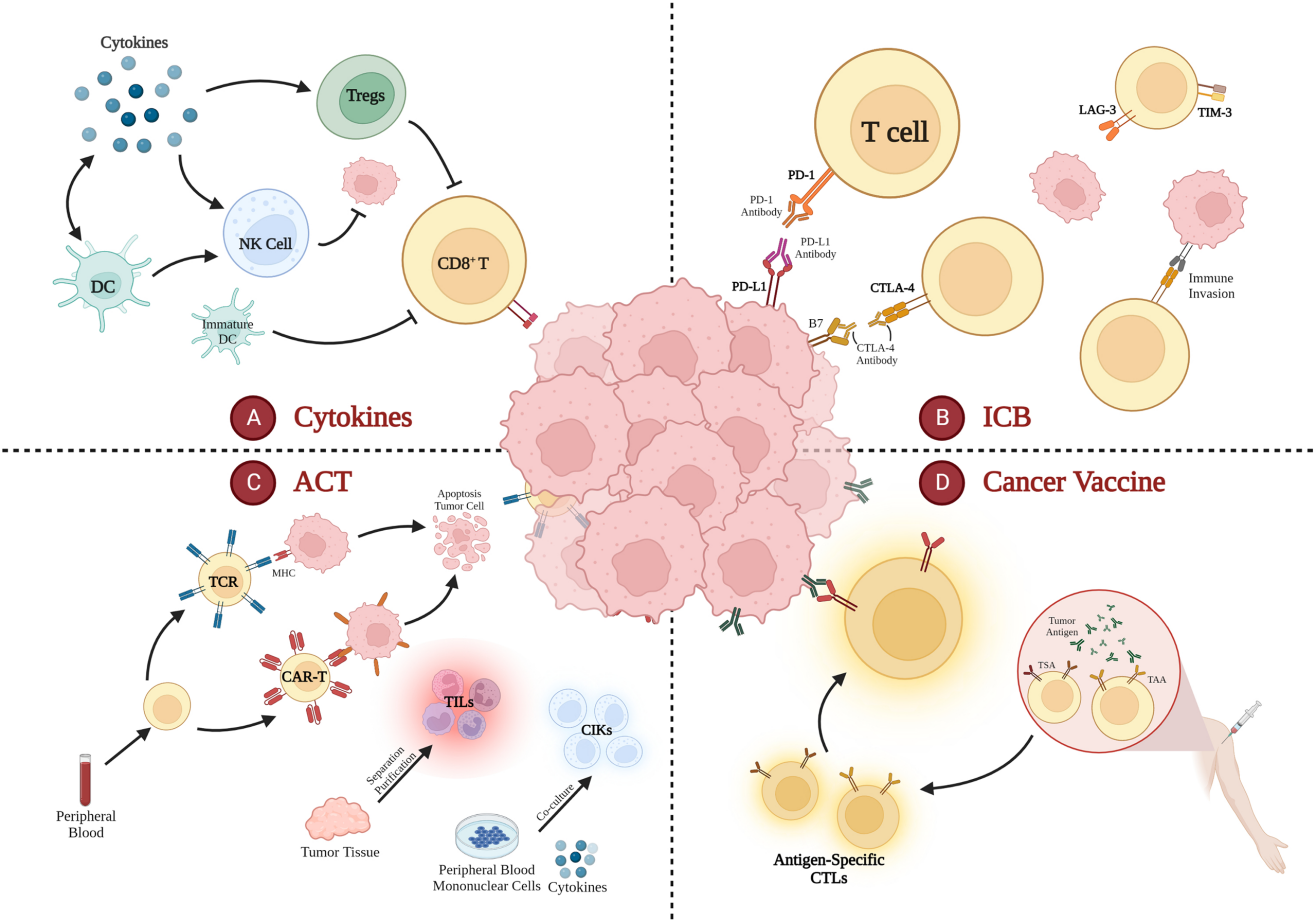
Applications of Immunotherapy in Cold CRC.

Immunotherapy has significantly altered the paradigm of partial cancer treatment due to its significant and durable therapeutic advantages. However, it has been discovered that colorectal cancer is one of the tumor types that does not react favorably to immunotherapy. Although the effectiveness of ICI has been demonstrated in dozens of clinical trials and with multiple FDA approvals, their response was restricted to relatively fewer patients with dMMR/MSI-H CRC. pMMR/MSS CRC, as a recognized cold cancer, is actually a real challenge for immunotherapy due to the heterogeneity of tumors and the complexity of the tumor microenvironment. In order to achieve “cold” to “hot” neoplastic transformation in MSS/pMMR CRC and subsequently overcome immunotherapy resistance, current immunotherapy-based research studies include ICIs, ACT, cancer vaccines, cytokines, and combinations of immunotherapies with chemotherapy, radiotherapy, targeted therapy, and other therapies (**Figure 2**). The discovery of novel medications, antibodies, and antigenic targets is still necessary to win the game.

## Author contributions

JY and JL had the idea for the article; CG and CJ performed the literature search and data analysis; JW and ZX drafted and critically revised the work. All authors contributed to the article and approved the submitted version.

## Conflict of interest

The authors declare that the research was conducted in the absence of any commercial or financial relationships that could be construed as a potential conflict of interest.

## Publisher’s note

All claims expressed in this article are solely those of the authors and do not necessarily represent those of their affiliated organizations, or those of the publisher, the editors and the reviewers. Any product that may be evaluated in this article, or claim that may be made by its manufacturer, is not guaranteed or endorsed by the publisher.
